# Management of shoulder dystocia: Number 7 – July 2022

**DOI:** 10.1055/s-0042-1755446

**Published:** 2022-08-08

**Authors:** Álvaro Luiz Lage Alves, Alexandre Massao Nozaki, Carla Betina Andreucci Polido, Roxana Knobel

**Affiliations:** 1 Hospital das Clínicas, Universidade Federal de Minas Gerais, Belo Horizonte, MG, Brazil; 2 Hospital Maternidade Interlagos, São Paulo, SP, Brazil; 3 Faculdade de Medicina, Universidade Federal de São Carlos, São Carlos, SP, Brazil; 4 Faculdade de Medicina, Universidade Federal de Santa Catarina, Florianópolis, SC, Brazil

## Key points

- Shoulder dystocia is a predominantly unpredictable and non-preventable event.
- The progressive incidence of obesity and diabetes has determined the contemporary increase in shoulder dystocia incidence.
- The main risk factors for shoulder dystocia are fetal macrosomia, diabetes mellitus, dystocia in the functional periods of labor and operative vaginal delivery.
- Clinical imaging and pelvimetry are not helpful in identifying women at higher risk for shoulder dystocia.
- Diagnosis and severity of shoulder dystocia are subjective. Failure of the head-shoulder maneuver and the turtle sign are the main diagnostic criteria. The need for multiple delivery maneuvers and the occurrence of maternal and/or neonatal injuries better evidence the severity of cases.
- Professionals involved in childbirth care must be prepared to recognize shoulder dystocia and immediately perform a sequence of maneuvers for its correction in a timely manner.
- Control of body weight and blood glucose levels is the main strategy likely to reduce the risk of shoulder dystocia.
- The most common serious maternal complications of shoulder dystocia are postpartum hemorrhage and complicated perineal lacerations.
- The most frequent neonatal complication of shoulder dystocia is transient brachial plexus palsy.
- Skills training and simulation improve the care and documentation of shoulder dystocia, promoting evidence-based management and reducing transient brachial plexus injuries.

## Recommendations

- In pregnancies with diabetes and estimated fetal weight above 4,500 grams (g) and in those without diabetes and estimated fetal weight above 5,000 g, cesarean section appears to prevent shoulder dystocia.
- In the prolongation of the second stage of labor in diabetic parturient women with an estimated fetal weight between 4,000 and 4,500 g, and in non-diabetic women with an estimated fetal weight between 4,500 and 5,000 g, cesarean section for the prevention of shoulder dystocia is also applicable.
- In the prolonged pelvic period of fetuses with estimated weight of more than 4,500 g, intrapartum cesarean section for prevention of shoulder dystocia is preferable to low operative vaginal delivery or forceps delivery. Similarly, operative vaginal delivery with the fetal head in the mid-pelvis should be avoided in fetuses estimated to weigh more than 4,000 g, and intrapartum cesarean section is indicated. In these situations, birth instrumentation should only be considered in the presence of experienced operators, through an individualized assessment of fetal position and size, history of previous deliveries and maternal habits.
- Induction of labor to prevent shoulder dystocia is indicated in pregnant women with gestational diabetes at 39 weeks and estimated fetal weight between 4,000 and 4,500 g. In pregnant women without diabetes, induction can also be offered at 39 weeks when the estimated fetal weight is between 4,000 and 5,000 g, but expectant management is also a reasonable alternative.
- A sequence of maneuvers that may vary according to the parturient woman’s position must be adopted in the treatment of shoulder dystocia. The first specific maneuver suggested when in lithotomy position is the McRoberts maneuver, which may be associated with external suprapubic pressure (Rubin I maneuver).
- In lithotomy position, when initial maneuvers fail, the main secondary maneuvers are Gaskin’s (all fours), posterior arm delivery and internal rotations (Rubin II, Woods screw and reverse Woods). Axillary traction for posterior shoulder delivery may also be a viable alternative.
- In upright positions, the recommended sequencing is to increase squat (modified McRobert maneuver), external suprapubic pressure, all-fours (Gaskin) position, internal maneuvers, and posterior arm delivery. Axillary traction of the non-impacted shoulder may also be an alternative.
- Rescue maneuvers for correction of shoulder dystocia include attempting to fracture the fetal clavicle and extracting the posterior shoulder with the aid of an axillary sling. In case of failure, last resort maneuvers are Zavanelli’s, abdominal rescue and symphysiotomy. Due to the maternal morbidity associated with the procedure, the symphysiotomy must be preceded by an assessment of risks and benefits and restricted to places where it is not possible to perform abdominal rescue given the lack of operating rooms.
- Documentation of shoulder dystocia should detail the care provided, the impacted fetal shoulder, time to resolution and associated complications. It is important for counseling patients and their caregivers, as well as for legal matters. Establishing effective communication with the parturient woman and her companions throughout the assistance is key, detailing the maneuvers performed and complications that occurred.
- Instituting active teaching protocols and methodologies provides evidence-based management and better performance of care teams in the treatment of shoulder dystocia.

## Background

Shoulder dystocia (SD) is an obstetric emergency characterized by the need for obstetric-surgical maneuvers in addition to the gentle downward traction exerted to loosen the fetal shoulders (head-shoulder maneuver). The event occurs due to impaction of the anterior fetal shoulder behind the maternal pubic symphysis after exteriorization of the cephalic pole. Simultaneous impaction of the posterior fetal shoulder on the sacral promontory may worsen dystocia. As most cases occur in the absence of prenatal or intrapartum risk factors, the event is often unpredictable and not preventable. Professionals involved in childbirth care must be prepared to recognize the event and immediately perform a sequence of maneuvers for its correction in a timely manner. 1 The main objective of treating shoulder dystocia is to prevent fetal asphyxia and permanent brachial palsy or death. Other neonatal injuries (fractures) and tract lacerations should also be avoided. To this end, the team’s organized performance and the quick and skillful sequencing of release maneuvers are essential. 1

## What is the epidemiological situation of shoulder dystocia?

The progressive incidence of obesity and diabetes, the risk factors for macrosomia and the increase in birth weight have determined the contemporary increase in shoulder dystocia. 2 The event occurs in 0.2%-3.0% of deliveries, and variations are related both to the subjectivity of diagnosis and the prevalence of macrosomia and diabetes in populations. 1 It is estimated that one newborn with hypoxic ischemic encephalopathy secondary to shoulder dystocia occurs in every 22,000 vaginal deliveries at term. 3

## What is the pathophysiology of shoulder dystocia and how should it be diagnosed?

**Figure 1.**
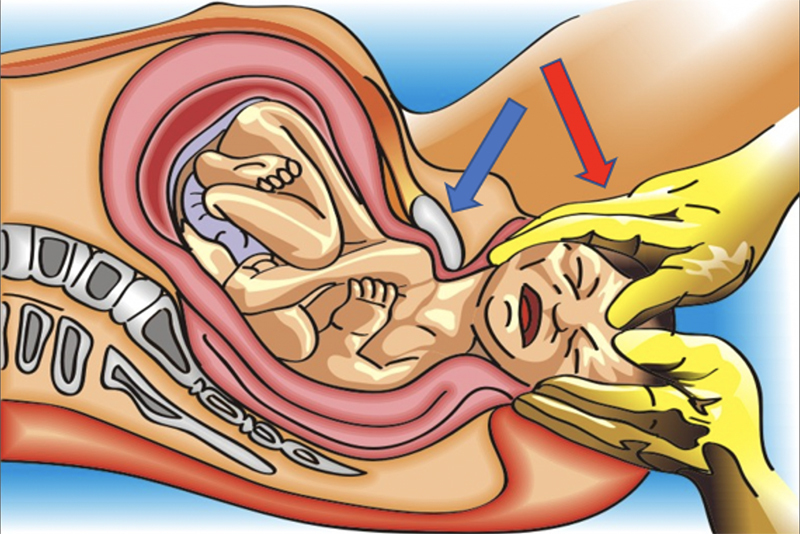
Head-shoulder maneuver (blue arrow: impaction of the anterior shoulder behind the pubic symphysis; red arrow: inferior pressure exerted on the cephalic pole in the head-shoulder maneuver). Source: Illustration by Felipe Lage Starling (authorized).

Shoulder dystocia occurs due to failure of the normal rotation of the shoulders to the oblique diameter at the moment of entry of the biparietal diameter into the pelvis. Rotational failure can occur as a result of resistance between the fetal skin and vaginal walls, premature delivery, or resistance from a proportionately larger thorax in relation to the biparietal diameter. Therefore, the anterior and posterior shoulders remain in the anteroposterior diameter of the maternal pelvis during descent and/or descend simultaneously, altering the physiological process in which the posterior shoulder descends in front of the anterior shoulder in an oblique diameter. 4 The subjectivity of the diagnosis and severity of shoulder dystocia is remarkable. The event is more evident when the head-shoulder maneuver fails ( Figure 1 ) and/or there is retraction of the fetal head towards the perineum, determined by the reverse traction of the impacted shoulder at the entrance of the pelvis (turtle sign). Retrospectively, the need for multiple maneuvers and the occurrence of maternal and/or neonatal injuries better evidence the severity of shoulder dystocia. More objective criteria, such as a greater than 60 second-interval between delivery of the head and shoulders, still need validation studies, both for the diagnosis and for the prediction of adverse neonatal outcomes. 5

## What are the main risk factors for shoulder dystocia and the strategies to reduce its incidence?

There are several risk factors for shoulder dystocia, both antepartum and intrapartum, and recognizing them allows discussing the possibility of elective cesarean sections and/or determining an individualized and attentive surveillance of the pelvic period. The main antepartum risk factors are fetal macrosomia, diabetes mellitus, past history of shoulder dystocia, post-term pregnancy, male fetal sex, maternal obesity/excessive gestational weight gain, and maternal age. Of these, the main one is fetal macrosomia. Maternal obesity, diabetes and advanced maternal age are also related to high birth weight. However, although shoulder dystocia progressively increases in births of fetuses weighing more than 4,000 g and neonatal morbidity and mortality due to shoulder dystocia also increase significantly among newborns weighing more than 4,500 g, fetal weight does not provide a good predictive value for shoulder dystocia. 6 Most macrosomic fetuses do not develop shoulder dystocia; approximately half cases of shoulder dystocia occur in newborns with birth weight of less than 4,000 g, and both ultrasound and clinical assessment (Leopold’s maneuvers, Johnson’s rule) have low sensitivity for estimating birth weight. 7

With regard to diabetes mellitus, in addition to its association with fetal macrosomia, the anthropometric measurements of fetuses of diabetic mothers favor the occurrence of shoulder dystocia. Fetuses of diabetic mothers with poor glycemic control, in addition to being large, are disproportionate due to centripetal deposits of body fat. Therefore, they have greater chest-head and shoulder-head ratios, increasing the risk for shoulder dystocia, even in those weighing less than 4,000 g. 8

Although the recurrence of shoulder dystocia is underestimated by subsequent choices for elective cesarean, its risk is at least 10%, and can reach 25%. Concomitant recurrence of fetal macrosomia is frequent. Birth weight gain secondary to advancing pregnancy explains the inherent risk of shoulder dystocia in post-term pregnancies. Male sex as a risk factor for shoulder dystocia seems to be explained by anthropometric measurements and a higher prevalence of macrosomia among men. Other risk factors that favor macrosomia and diabetes, and consequently shoulder dystocia, are obesity and excessive maternal weight gain. Contemporary maternal demographics, with a higher prevalence of pregnant women aged over 35 years, constitute a risk for shoulder dystocia, in association with a higher prevalence of diabetes, overweight and multiparity among these patients. 9 10 11

The main intrapartum risk factors are dystocia in the functional stages of labor (dilation and pelvic) and operative vaginal delivery. In isolation, pelvic period abnormalities (prolonged pelvic period and secondary arrest of dilation) are not useful predictors of shoulder dystocia. However, when combined with estimated fetal weight greater than 4,000 g and operative vaginal delivery, they are associated with a higher incidence of shoulder dystocia. 12

Operative vaginal delivery is related to shoulder dystocia. However, there is no evidence that fetal traction performed by forceps or vacuum extractor increases the risk of shoulder impaction or that previous poor shoulder positioning inhibits fetal descent, resulting in the need for labor instrumentation. Apparently, the risks for shoulder dystocia are similar for the forceps and the vacuum extractor. 13

Despite the diversity of risk factors for shoulder dystocia, few of them are modifiable. Control of body weight before and during pregnancy and control of glycemic levels among diabetic women are the main strategies likely to reduce risk. Therefore, dietary and lifestyle interventions can reduce the rates of macrosomic fetuses and shoulder dystocia, especially among diabetic pregnant women. 14

## Which conducts or procedures are efficient to prevent shoulder dystocia?

Since shoulder dystocia predominantly occurs in parturient women with normal pelvic dimensions, imaging tests and clinical pelvimetry are not useful in identifying women at higher risk, except in rare cases of severe pelvic or fetal abnormalities. 6

Mildly abnormal fetal biometry is not predictive of shoulder dystocia. The various fetal biometric parameters (difference between abdominal and biparietal diameters, chest circumference, head circumference/abdominal circumference ratio, femoral length/abdominal circumference ratio, humerus-spinal distance, cheek-to-cheek diameter, shoulder width) were either not tested in large prospective studies or have not proved to be useful to predict shoulder dystocia. 15

Even though the consensus is that planned cesarean section for macrosomic fetuses is appropriate to reduce shoulder dystocia, this approach has not proven advantageous, since most cases of shoulder dystocia and brachial plexus injury cannot be predicted or avoided, leading to unjustified elevation of cesarean rates. However, for fetuses of diabetic mothers estimated to weigh more than 4,500 g, as well as for fetuses weighing more than 5,000 g in the absence of diabetes, assessed within a week until delivery, cesarean section appears to be able to reduce shoulder dystocia and associated morbidity. 1

The principle of cesarean section to prevent shoulder dystocia also applies when the second stage of labor is prolonged (primiparous women with analgesia: four hours; primiparous women without analgesia: three hours; multiparous women with analgesia: three hours; multiparous women without analgesia: two hours) in diabetic parturient women with an estimated fetal weight between 4,000 and 4,500 g, as well as in non-diabetic parturient women with an estimated fetal weight between 4,500 and 5,000 g. Intrapartum caesarean rather than low operative or forceps vaginal delivery is suggested for fetuses estimated to weigh over 4,500 g and prolonged pelvic period. Intrapartum cesarean section instead of operative vaginal delivery in the middle pelvis is also suggested for fetuses with an estimated weight above 4,000 g, in the presence of a prolonged pelvic period. However, operative vaginal delivery in these situations can be considered in the presence of experienced operators, individualized assessment of fetal position and size, history of previous deliveries and maternal habits. 16

The induction of labor in macrosomic fetuses as a measure to prevent shoulder dystocia is also limited, given the low accuracy of fetal weight estimation methods, the unfavorable relationship between the number of inductions necessary to prevent adverse outcomes, maternal and neonatal consequences related to the induction process, and the lack of scientific evidence on the effectiveness of this conduct. Induction of labor can be offered to pregnant women without diabetes at 39 weeks and estimated fetal weight between 4,000 and 5,000 g, but expectant management is a reasonable alternative. Induction at 37 or 38 weeks in this situation is not recommended, as although it potentially promotes a greater reduction in shoulder dystocia, it leads to an increase in common neonatal morbidities (hyperbilirubinemia, respiratory problems). 17 For women with pregestational diabetes, the definition of the moment of delivery should be based on maternal risks and other perinatal risks associated with the disease, with little benefit in maintaining the pregnancy beyond 39 weeks and the need for premature interruption in the face of vasculopathy and/or poor glycemic control. Among women with gestational diabetes and estimated fetal weight between 4,000 and 4,500 g, induction of labor performed at 39 weeks potentially reduces shoulder dystocia, with maternal and other neonatal risks (respiratory distress, intensive care) at a lower incidence. Fetal weight, glycemic control throughout pregnancy, birth weight and results of previous deliveries, as well as the parturient woman’s physical characteristics (height, weight, body mass index, pelvimetry) should be evaluated. 18 Labor induction at 41 week of gestation reduces the birth of newborns weighing more than 4,000 g, with a potential reduction in shoulder dystocia. 19

In patients with a history of shoulder dystocia, especially those with severe neonatal injury, the potential risk of recurrence (10% or more) and the risk factors of the current pregnancy (estimated fetal weight, blood glucose) should be considered when deciding on the route of childbirth. 9

## How should the initial management of shoulder dystocia be?

Shoulder dystocia management is aimed at the complete and safe fetal delivery, before asphyxia and cortical injury resulting from umbilical cord compression and impediment of inspiration, avoiding peripheral neurological injuries or other fetal and/or maternal trauma. The time limit that precedes the increase in the risk of injury from asphyxia is five minutes, which imposes the instant need for organization and effective team action. 20

Immediately after the suspicion of shoulder dystocia, the parturient woman and her companion must be communicated and the following actions must be implemented: request for help from other professionals (assistant and obstetrical nurses, obstetricians, pediatricians and anesthesiologists); documentation of the moment of diagnosis and timing of assistance; orientation contrary to voluntary pushing. The following conducts are essential:

1. Do not exert excessive traction to release the shoulders and do not put pressure on the uterine fundus, as these actions are associated with stretching the brachial plexus, worsening impaction and uterine rupture.
2. Do not cut the umbilical cord before releasing the shoulders, as this action does not contribute to resolution of shoulder dystocia and further reduces the oxygenation of the fetus. If present, umbilical cord loops must be released without sectioning.
3. Avoid performing an episiotomy, as the procedure does not resolve shoulder dystocia that results from bone impaction. However, given the need for internal maneuvers, episiotomy may be necessary in cases where perineal resistance makes it difficult to perform the maneuvers. Bladder catheterization may also be necessary.
4. Promote efficient communication among members of the care team, who must receive clear and objective information on the actions taken and the outcomes, avoiding unnecessary repetition of maneuvers and optimizing management in a timely manner. 21

Evidence on the effectiveness and sequencing of the various maneuvers is scarce. There is no definition about which maneuver is superior to another, nor about what is the ideal sequence of maneuvers. The use of maneuvers attempts to resolve shoulder dystocia through three mechanisms:

1. Enlargement of maternal pelvic dimensions.
2. Reduction of fetal biacromial diameter through adduction of the shoulders or shift of the posterior arm.
3. Modification in the relationship between the biacromial diameter of the fetus and the maternal bony pelvis by rotating the fetal trunk to the oblique diameter of the pelvis (wider) and releasing the anterior shoulder behind the pubic symphysis or delivering the posterior arm and/or shoulder. 21

## How should the patient be managed in the lithotomy position?

In lithotomy, the parturient woman must be positioned with the buttocks close to the edge of the bed or delivery stretcher. The traction to release the shoulders must be axial and aligned with the fetal cervicothoracic spine in a descending component along a vector that does not exceed 45° below the horizontal plane of the parturient woman. Failure of the head-shoulder maneuver performed with habitual force is indicative of shoulder dystocia. Therefore, the perception of excessive force to release the shoulder indicates the need for specific maneuvers. 1

**Figure 2.**
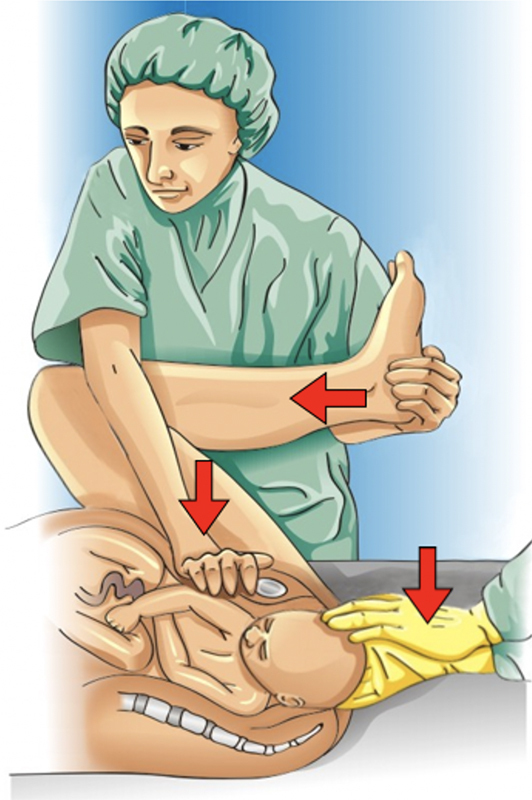
McRobert and Rubin I maneuvers. Source: Illustration by Felipe Lage Starling (authorized).

**Figure 3.**
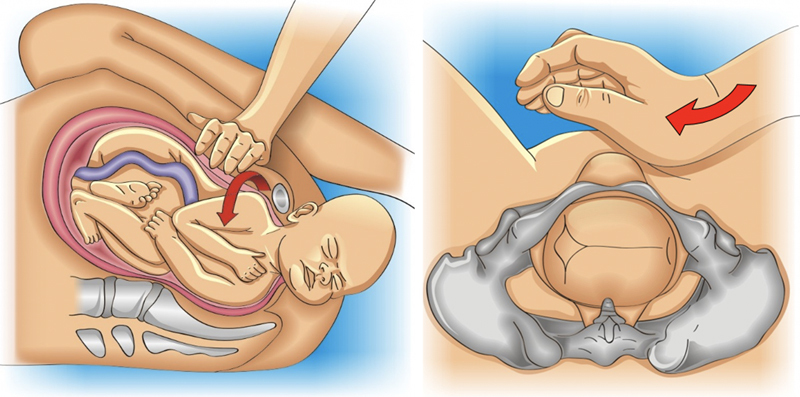
Rubin I maneuver. Source: Illustration by Felipe Lage Starling (authorized).

The suggestion for the first specific maneuver to be applied is the McRobert maneuver, which can be associated with the Rubin I maneuver ( Figures 2 and 3 ). These maneuvers are efficient and less invasive. In the McRobert maneuver, the lower limbs are flexed against the abdomen (hyperflexion of the legs and thighs), and should be previously dislocated when accommodated in the legs of the stretcher. This position promotes vertical alignment of the maternal pelvis with cephalad rotation of the pubis, reduction of lumbar lordosis, rectification of the promontory, rotation of the pubic symphysis on the impacted shoulder, flexion of the fetal spine, and fall of the posterior shoulder into the sacral concavity. In addition, there is an increase and redirection of the expulsive force that becomes perpendicular to the output plane. The Rubin I maneuver performed simultaneously with the McRobert maneuver optimizes shoulder release through its adduction. In patients with significant obesity, this step can be omitted. The maneuver is performed by an assistant who, positioned on the side of the fetal back, performs suprapubic compression in an inferomedial direction. Compression should be performed with the hands flat positioned similarly to cardiac massage. Under command of the obstetrician who performs the lower traction on the fetal head, the Rubin I maneuver should be started immediately before the head-shoulder maneuver. As soon as the suprapubic compression begins, the head is pulled downwards, promoting delivery of the shoulder. 1 22

**Figure 4.**
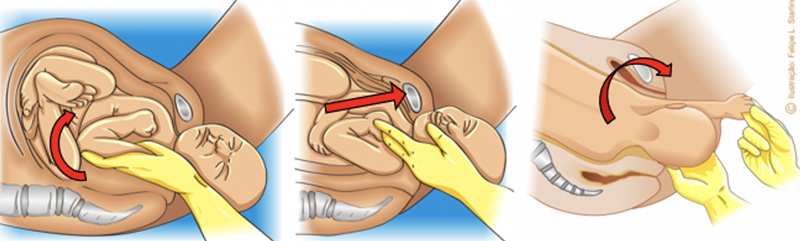
Release of the posterior arm by the Jacquemier maneuver. Source: Illustration by Felipe Lage Starling (authorized).

**Figure 5.**
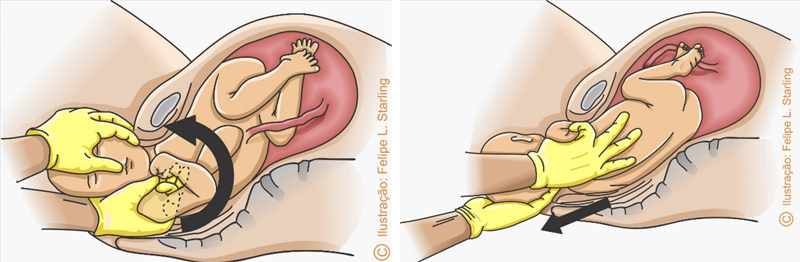
Delivery of the posterior arm by the shrug maneuver. Source: Illustration by Felipe Lage Starling (authorized).

In case the McRobert and Rubin I maneuvers fail, the sequencing of maneuvers must progress to complete delivery of the posterior arm or release of the posterior shoulder. Although more invasive, the maneuver for complete delivery of the posterior arm (Jacquemier maneuver) has a high success rate in resolving shoulder dystocia. 1 22 The obstetrician must grasp the posterior arm in front of the fetal thorax. Therefore, if the fetal back is facing the right maternal side, the operator’s right hand must perform the maneuver on the left side of the maternal pelvis, and vice versa. The maneuver is performed in three steps and an episiotomy may be necessary to facilitate the procedure. First, the hand is introduced into the vagina, progresses through the sacral hollow, and grasps the posterior arm with the fingers positioned parallel to the humerus. In a second step, the arm is moved in front of the fetal thorax. If the fetal arm is extended, it is necessary to apply pressure on the antecubital fossa to optimize displacement. In a third step, the forearm and hand are grasped and pulled out of the vagina, passing in front of the fetal thorax, promoting anterior rotation of the fetal trunk and subsequently, the delivery of the posterior forearm, arm and shoulder through the anterior space of the maternal pelvis ( Figure 4 ). Delivery of the posterior arm promotes a reduction of 2 to 3 cm in the biacromial diameter, transforming it into an axilloacromial diameter of 10-11 cm, enabling the resolution of shoulder dystocia. 3 Given the difficulty to grasp and anteriorly shift the posterior arm (arm extended or positioned behind the fetal back), the Shrug maneuver is an efficient alternative to the Jacquemier maneuver. In this maneuver, the operator’s hand grasps the fetal posterior axilla, interlacing the thumb and index fingers in the axillary cavus. The axilla is shifted towards the fetal head, placing the posterior shoulder at a lower level than the pubic symphysis. Simultaneously, the other hand holds the fetal head. The head and shoulder are rotated together through 180° toward the fetal face, delivering the posterior shoulder, anterior to the pelvis. Located posteriorly after rotation of the fetal trunk, the anterior shoulder is the last to be delivered ( Figure 5 ). 23

**Figure 6.**
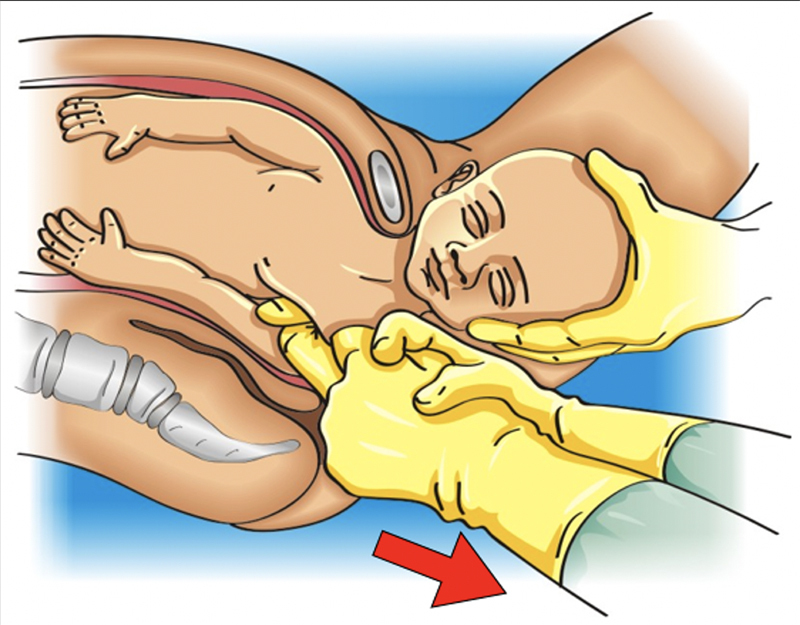
Delivery of the posterior shoulder by the Menticoglou maneuver. Source: Illustration by Felipe Lage Starling (authorized).

If delivery of the posterior arm fails, given the difficulty in reaching the elbow or forearm, another strategy is to perform axillary traction for descent or delivery of the posterior shoulder (Menticoglou maneuver). 24 The maneuver is performed by interlacing the middle fingers of each operator’s hand in the posterior axilla of the fetus. While an assistant flexes the fetal head toward the impacted anterior shoulder, the operator inserts the middle finger of his left hand into the right side of the maternal pelvis and the right middle finger into the contralateral side. The fingers are interlaced in the fetal axillary cavus and an inferior traction is performed along the sacral curvature ( Figure 6 ). This maneuver also facilitates a new attempt at complete delivery of the posterior arm or the subsequent execution of an internal rotation maneuver for delivery of the impacted anterior shoulder. This maneuver also often promotes spontaneous delivery of the anterior shoulder. 24

An alternative approach for delivery of the posterior shoulder is to perform a single-handed underarm grasp and lower shoulder traction. In this situation, the index finger will wrap around the axilla along the fetal back and the thumb will slide anteriorly to the shoulder. Fingertips should touch at the fetal axillary cavus. Subsequently, lower traction is performed. 25

If the initial approach fails, secondary maneuvers must be instituted. The main ones are the Gaskin maneuver and the internal rotation maneuvers (Rubin II, Woods screw and reverse Woods). 1 22

**Figure 7.**
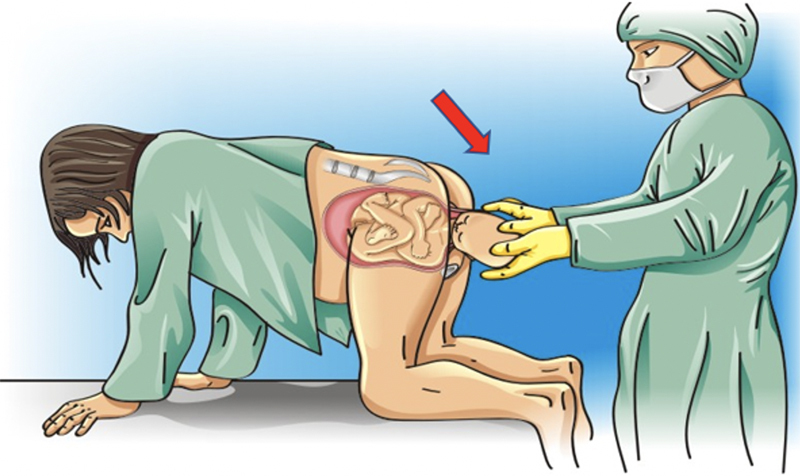
Gaskin meneuver. Source: Illustration by Felipe Lage Starling (authorized).

**Figure 8.**
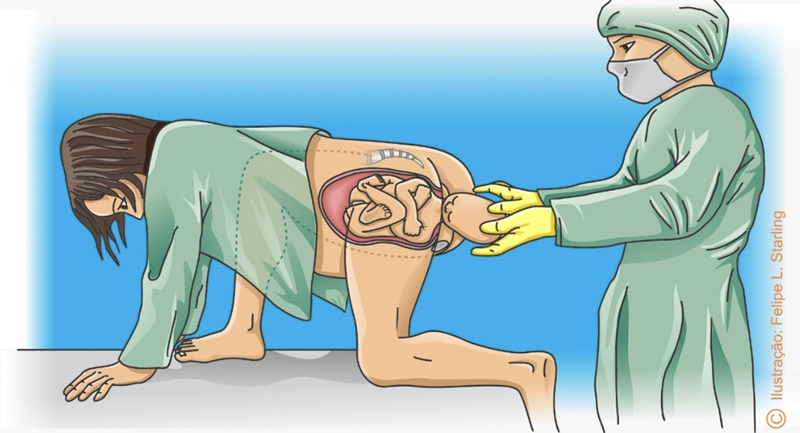
“Sprint start” position. Source: Illustration by Felipe Lage Starling (authorized).

In the Gaskin maneuver, the parturient woman is positioned in “all fours” (hands and knees) ( Figure 7 ). Alternatively, the “sprint start” position can be adopted, in which the lower limb homolateral to the fetal back will be flexed and displaced anteriorly to the maternal pelvis, while the other leg remains extended posteriorly ( Figure 8 ). These positions expand the space in the sacral cavity and benefit from gravity (verticalization of the maternal trunk). Traction can be performed downward (posterior shoulder delivery) or upward (anterior shoulder delivery). These maneuvers are interesting options given their high efficacy and low morbidity, especially for parturient women without analgesia and/or assisted in delivery beds. They may also optionally precede the attempt to deliver the posterior arm or shoulder, which are technically more difficult maneuvers. 26

**Figure 9.**
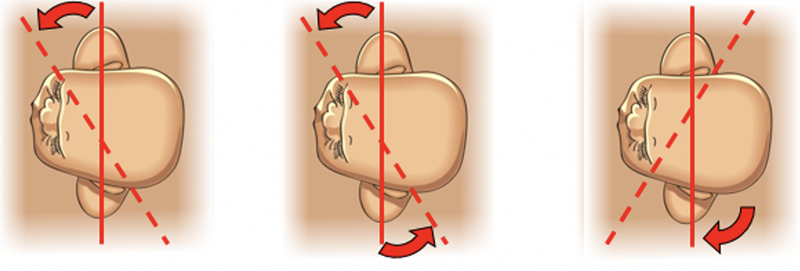
Sequencing of internal rotational maneuvers of Rubin II, Woods screw and reverse Woods. Source: Illustration by Felipe Lage Starling (authorized).

Internal rotational maneuvers must be applied in sequence ( Figure 9 ). The first attempt should be to adduct the impacted anterior shoulder using the Rubin II maneuver. The operator’s hand performing the maneuver must be on the side corresponding to the fetal back. The hand should be introduced through the sacral hollow homolateral to the fetal back, be shifted upward and placed behind the impacted anterior shoulder to promote its adduction. The objective is to shift the shoulder to the oblique diameter of the pelvis that has wider dimensions. In the face of failure, the hand should be kept behind the anterior fetal shoulder while the other hand is introduced into the contralateral sacral hollow and placed in front of the posterior shoulder. Thus, the upper hand performs posterior adduction compression of the impacted shoulder while simultaneously, the lower hand anteriorly compresses the posterior shoulder, abducting it and optimizing the attempt to rotate the biacromial diameter to the oblique diameter of the pelvis. The maneuver performed in front of the posterior shoulder is called Woods screw. If this second attempt is unsuccessful, the operator’s lower hand is removed from the genital tract and the upper hand is shifted inferiorly, placing it behind the posterior shoulder to perform the reverse Woods screw maneuver, which also promotes 180° internal rotation of the fetal back, reversing the fetal shoulders to an oblique position of delivery. 1 22 27

The HELPERR mnemonic is proposed for professional training in the management of shoulder dystocia in the lithotomic position ( Chart 1 ). After warning the parturient woman, providing help and an anesthesiologist, the proposed sequencing includes the McRoberts and Rubin I maneuvers, assessment of the need for episiotomy, removal of the posterior arm, internal maneuvers and, finally, changing the position to all-fours. 28

**Chart 1.** HELPERR mnemonic for sequencing maneuvers in the treatment of shoulder dystocia

H	Call for Help: obstetrics, neonatology, anesthesia, warn the patient
E	Evaluate for Episiotomy
L	Legs flexed: McRoberts maneuver
P	suprapubic Pressure (Rubin I maneuver)
E	Enter maneuvers ((Rubin II, Woods screw, reverse Woods)
R	Remove the posterior arm
R	Roll the patient (all fours, Gaskin maneuver)

If the shoulder dystocia is not resolved after attempting the initial and secondary maneuvers described above, assistance must progress to last resort maneuvers (rescue). 1 22

## How should shoulder dystocia be managed in vertical positions?

The obvious benefit of freedom of position and vertical positions has contributed to the greater adoption of squatting positions, Gaskin and support benches during assistance in the second stage of labor. In these situations, shoulder dystocia can be solved by means of another sequence of maneuvers in order to avoid additional loss of time. 29

**Figure 10.**
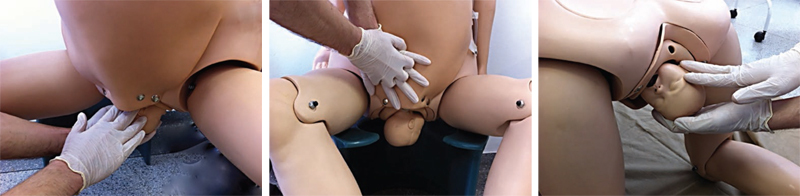
Initial sequence of maneuvers in the A SAÍDA mnemonic. Augment the squat (modified McRoberts maneuver); suprapubic pressure and alter the parturient woman’s position (all-fours). Source: Adapted from Amorim et al. (2013). 29

**Figure 11.**
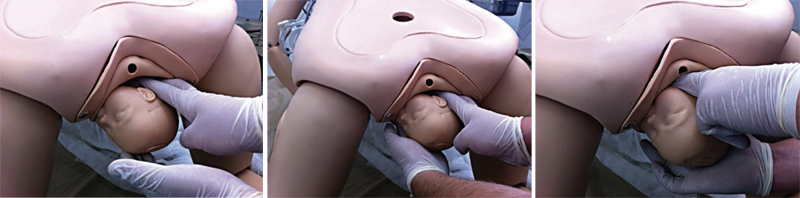
Intermediate sequence of maneuvers in the A SAÍDA mnemonic. Rubin II, Woods screw and reverse Woods maneuvers. Source: Adapted from Amorim et al. (2013). 29

**Figure 12.**
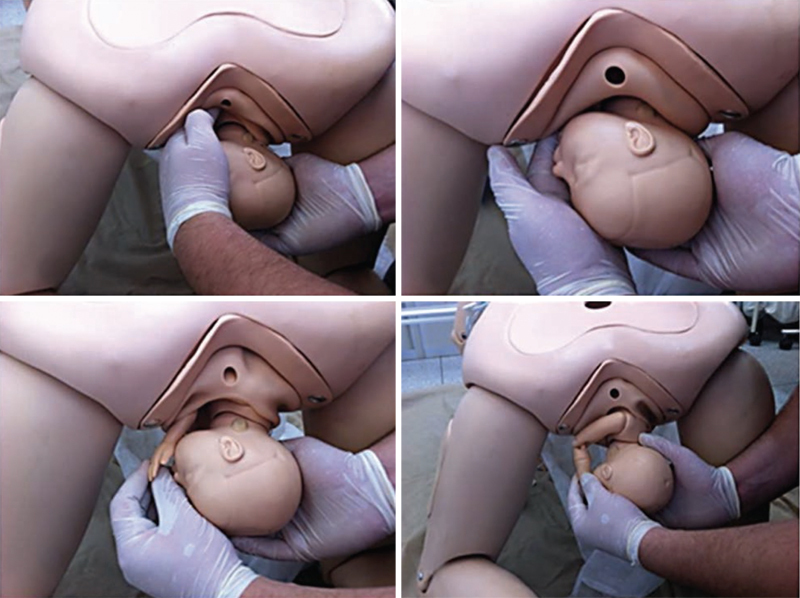
Final sequence of maneuvers in the A SAÍDA mnemonic. Delivery of posterior arm in all-fours position (Gaskin). Source: Adapted from Amorim et al. (2013). 29

The mnemonic A SAÍDA ( Chart 2 ) is proposed for professional training in the management of shoulder dystocia in parturient women in an upright position, free from a stretcher or operating table. The sequencing starts with an increase in the maternal squat that will promote hyperflexion of the lower limbs, widening the functional diameter of the pelvis, similarly to the McRoberts maneuver (modified McRoberts maneuver). If hyperflexion alone is not sufficient to resolve shoulder dystocia, the next step is to exert external suprapubic pressure, keeping the parturient in an extended squat. Pressure is performed by placing the hands on the maternal lower abdomen, as in the Rubin I maneuver. In the event of failure, the subsequent step is to change the parturient woman’s position to all-fours (Gaskin or “sprint start” positions). If resolution does not occur, the parturient woman will be kept in all-fours for a subsequent attempt at internal maneuvers. The suggested sequence is the same as for the lithotomy position: Rubin II maneuvers, Woods screw and reverse Woods screw. The technique adopted for executing the maneuvers will also be practically the same. What changes is that fetal shoulders are inverted given the change in maternal position, that is, the anterior shoulder impacted on the maternal pubis is located inferiorly and the posterior shoulder is superior. Thus, compared to the lithotomy position, maneuvers are performed on the opposite shoulders. Then, the Rubin II maneuver is performed behind the posterior shoulder, now positioned superiorly; the Woods screw is performed by adding pressure with the other hand in front of the anterior shoulder, positioned inferiorly; and the reverse Woods maneuver is performed inferiorly, behind the anterior shoulder, after the operator moves the hand inferiorly. Faced with the failure of sequencing the internal maneuvers, the next attempt is the delivery of the posterior arm (Jacquemier maneuver), here located superiorly. The operator’s hand penetrates superiorly through the sacral hollow and grasps and shifts the posterior arm anteriorly in the fetal thorax. Then, the operator moves his hand to grasp the fetal hand, performing an inferior traction on it and promoting the rotation of the fetal body and the orderly release of the posterior hand, arm and shoulder. 29 Figures 10 , 11 and 12 illustrate the sequencing of maneuvers according to the A SAÍDA mnemonic.

**Chart 2.** The A SAÍDA mnemonic for sequencing maneuvers in the treatment of shoulder dystocia in vertical positions

A	Ask for help; Acquaint the mother; Anesthetist ready; Augment the squat (modified McRoberts)
S	Suprapubic pressure
A	Alter the position to all fours (Gaskin maneuver)
Í	Internal maneuvers (Rubin II, Woods screw, reversal Woods)
D	Deliver the posterior arm
A	Assess the need for rescue maneuvers

If resolution with this sequencing does not occur, assistance must progress to last resort maneuvers (rescue). 29

## What other sequences of maneuvers can be adopted in the treatment of shoulder dystocia?

A situational approach can be adopted according to the maternal position at the time of shoulder dystocia and with movements of the pelvis. In the sequencing proposed by Harder (2005), 30 , movements to promote counternutation of the sacrum are performed before internal maneuvers, lifting the lumbosacral joint against gravity and increasing the anteroposterior diameter of the posterior pelvis. Combined with body movement, sacral counternutation helps to resolve shoulder dystocia. With the parturient in lithotomy, the buttocks are moved close to the edge of the delivery bed (or stretcher) and the lower limbs are released below the level of the pelvis, in the Crouzat-Walcher position, expanding the upper strait. Soon after, the parturient woman is positioned in McRoberts. This same sacral counternutation movement is also proposed for other positions adopted during childbirth (Sims, knees, Gaskin). 30 Tully’s protocol (2012) 31 proposes that maneuvers are initiated with the parturient woman in the Gaskin position or in “sprint start”. The Rubin II maneuver is performed by rotating the biacromial diameter of the fetus to the oblique diameter of the maternal pelvis and enlarging the space for insertion of the hand that will remove the posterior arm. 31

## What are the last resort maneuvers (rescue) in the treatment of shoulder dystocia?

**Figure 13.**
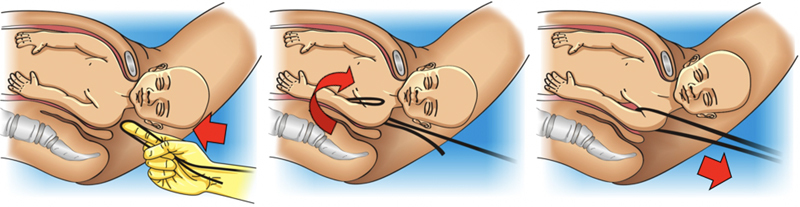
Delivery of posterior shoulder with the aid of a sling. Source: Illustration by Felipe Lage Starling (authorized).

Fracture of the clavicle and delivery of the posterior shoulder with the aid of a sling are maneuvers that can be attempted immediately before the classic last resort maneuvers (Zavanelli, abdominal rescue and symphysiotomy). However, they do not fit into the initial management of shoulder dystocia described above, as they are associated with increased neonatal morbidity. 22 32 The anterior clavicle can be intentionally fractured, reducing the biacromial diameter and releasing the impacted shoulder. In this technique, the operator must use the fingers to pull the clavicle out until it breaks. The procedure can be technically difficult and associated with injuries to the underlying fetal vascular and pulmonary structures. However, it is a less morbid procedure than last resort maneuvers. 22 A sling applied to the posterior axilla can help in delivery of the posterior shoulder. Humeral fracture appears to be the main associated neonatal morbidity. A #12 or #14 urinary catheter (or a suction catheter) is looped over the tip of the index finger that will be placed behind the posterior shoulder. The loop is pushed behind the posterior axilla until it is retrieved by the other index finger, which is introduced into the contralateral side of the pelvis, anterior to the fetal thorax. The loop is subsequently unfolded, forming a sling around the posterior shoulder. The ends of the sling are grasped and a moderate lower traction is performed until delivery of posterior shoulder ( Figure 13 ). The sling can also be used to promote a 180° rotation of the shoulders, with the aid of a counterpressure exerted behind the anterior shoulder. 32

The last resort maneuvers (Zavanelli, abdominal rescue and symphysiotomy) present greater morbidity, and their risks and benefits must be evaluated considering the fetal conditions and local possibilities for performing interventions and treating complications. Muscle (sedation, general anesthesia) and uterine relaxation optimize the success of these maneuvers. Terbutaline (0.25 mg, subcutaneously) and nitroglycerin (50 mcg every minute until relaxation is achieved; maximum dose 250 mcg) are recommended. 33

**Figure 14.**
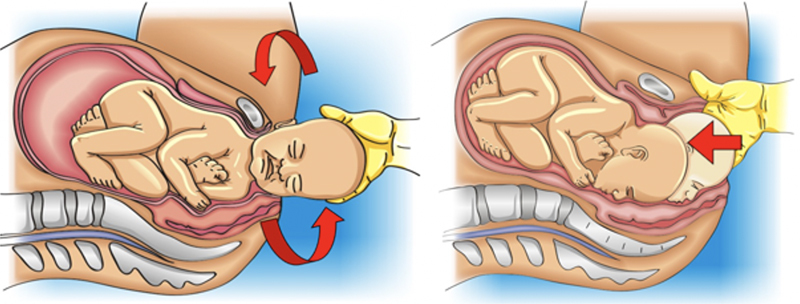
Zavanelli maneuver. Source: Illustration by Felipe Lage Starling (authorized).

The Zavanelli maneuver (Gunn-Zavanelli-O’Leary) repositions the fetal head in the pelvis for a subsequent cesarean section ( Figure 14 ). The initial step is the reversal of external rotation, positioning the occiput anteriorly. Next, the head is flexed and, by means of firm pressure exerted with the palm of one hand, it is pushed superiorly as high as possible into the vagina. The other hand can simultaneously depress the perineum, relieving umbilical cord compression and facilitating vaginal repositioning of the head. 33

In abdominal rescue, the parturient woman undergoes laparotomy and hysterotomy for transabdominal manual rotation of the anterior shoulder. After rotating the biacromial diameter to the oblique diameter of the pelvis, fetal extraction is performed vaginally by another operator. 34

Although it can save lives, symphysiotomy should be an exceptional maneuver given the lack of evidence regarding its efficacy and safety, and the possible associated morbidities, particularly pelvic instability. The surgical division of the pubic symphysis cartilage widens the pelvic opening, clearing the shoulder. Although it can solve the shoulder dystocia, it should only be performed when other maneuvers fail and in places where it is not possible to perform abdominal rescue due to the lack of operating rooms. The technique is performed under local anesthesia with the parturient woman in lithotomy and lower limbs abducted. After bladder catheterization, the anesthetic should be infiltrated into the skin and subcutaneous tissue overlying the pubic cartilage. The operator deviates the urethra laterally with one hand and makes a 1-3 cm incision with a scalpel blade. The incision must be sufficient to separate the pubic branches and release the impacted shoulder. Therefore, it is not necessary to incise the full thickness of the cartilage. After the procedure, absolute rest is recommended for two days, followed by progressive mobilization. Lower limb abduction should be avoided for 7-10 days. 35

## What are the main maternal and neonatal complications associated with shoulder dystocia?

The most common serious maternal complications of shoulder dystocia are postpartum hemorrhage (uterine atony and tract injuries) and complicated perineal lacerations. Other complications include pubic symphysis diastasis, urinary tract injuries (urethra and bladder), and transient lateral femoral cutaneous neuropathy secondary to the McRoberts maneuver. Last resort maneuvers may be more associated with uterine rupture, pubic symphysis diastasis and urinary tract injuries. 36

Neonatal injuries affect 5% of cases of shoulder dystocia. They can occur even when treatment is properly instituted. Continuing fetal head descent simultaneously with shoulder impaction stretches the brachial plexus nerves, with potential aggravation of injuries determined by the delivery maneuvers performed. Additionally, compression of umbilical cord and fetal neck vessels and excessive vagal stimulation are events that result in neonatal asphyxia. The most frequent neonatal complication is brachial plexus injury. Brachial plexus strain is significantly more frequent when three or more maneuvers are performed. Injuries in C5 and C6 roots or in C5, C6 and C7 (Duchenne-Erb palsy) have a better prognosis and recover within six months in more than half of infants. On the other hand, injuries involving all roots from C5 to T1 are restored in approximately 14% of cases. 37

Although shoulder dystocia and excessive operator force are important risk factors for brachial plexus strain, these injuries often occur in the absence of shoulder impaction, in cesarean sections, or in association with prenatal injuries. Therefore, it seems that propulsive forces, fetal position and maternal pushing may be sufficient to cause injurious traction of the brachial plexus. 38 Other serious neonatal complications are clavicle and humerus fractures, pneumothorax, hypoxic-ischemic encephalopathy and neonatal death. Rarer neonatal complications include diaphragmatic paralysis, Horner’s syndrome (oculosympathetic palsy), facial nerve injury, spiral radial fracture, and laryngeal nerve palsy. 36

## What are the main aspects in the documentation of shoulder dystocia?

Recording the events related to shoulder dystocia with details of the care provided and complications is strictly recommended. Proper event documentation is important for advising patients and their caregivers of future risks, as well as for legal matters. The use of standardized forms offers better documentation. 39

The team members who participated in the assistance, as well as the time spent until each one of them arrived at the birth scene must be in the documentation. The description must detail which shoulder was impacted and in which shoulder the delivery maneuvers were performed. Maneuvers must be described according to the sequencing that took place. The time spent in each maneuver should be detailed, as well as the time interval until resolution. 39

Estimated blood loss, birth canal review details, Apgar score, umbilical cord pH, and neonatal assessment should also be reported. 39

## What is the impact of skills training and simulation in the treatment of shoulder dystocia?

Skills training and simulation provide improvements in the use of maneuvers, communication between team members and event documentation. The use of protocols and active teaching methodologies promotes evidence-based management and the reduction of transient brachial plexus injuries. 40

## Final considerations

The unpredictability and potential severity of shoulder dystocia, as well as the limitation of time for its resolution without sequelae, make this event one of the most challenging of obstetric emergencies, requiring joint and organized action from caregivers, with rapid and skillful institution of specific obstetric-surgical maneuvers. The contemporary epidemiological scenario of shoulder dystocia is marked by an increase in its incidence, determined by the high prevalence of obesity and diabetes during pregnancy. In view of these aspects, we can say that all professionals who assist childbirth must be trained for the rapid recognition and resolution of this event, aiming to prevent asphyxia and neonatal death, permanent brachial palsy and associated maternal complications. Since skills training and simulation optimize the clinical resolution and documentation of shoulder dystocia, instituting protocols and active teaching methodologies is essential to provide evidence-based management and better performance of care teams in coping with this “obstetric nightmare”.

National Commission Specialized in Obstetric Emergencies of the Brazilian Federation of Gynecology and Obstetrics Associations (Febrasgo)

President:

Álvaro Luiz Lage Alves

Members:

Gabriel Costa Osanan

Samira El Maerrawi Tebecherane Haddad

Adriana Amorim Francisco

Alexandre Massao Nozaki

Brena Carvalho Pinto de Melo

Breno José Acauan Filho

Carla Betina Andreucci Polido

Eduardo Cordioli

Frederico José Amedée Peret

Gilberto Nagahama

Laíses Braga Vieira

Lucas Barbosa da Silva

Marcelo Guimarães Rodrigues

Rodrigo Dias Nunes

Roxana Knobel
